# Fine Characterization of a Resistance Phenotype by Analyzing TuYV-*Myzus persicae*-Rapeseed Interactions

**DOI:** 10.3390/plants10020317

**Published:** 2021-02-06

**Authors:** Marlène Souquet, Elodie Pichon, Thomas Armand, Emmanuel Jacquot

**Affiliations:** PHIM Plant Health Institute Montpellier, University of Montpellier, INRAE, CIRAD, Institut Agro, IRD, CEDEX 5, 34398 Montpellier, France; marlene.souquet@inrae.fr (M.S.); Elodie.pichon@inra.fr (E.P.); thomas.armand@etudiants.purpan.fr (T.A.)

**Keywords:** viral disease, rapeseed, aphid, resistance, transmission, turnip yellows virus

## Abstract

Turnip yellows virus (TuYV), transmitted by *Myzus persicae*, can be controlled in rapeseed fields by insecticide treatments. However, the recent ban of the neonicotinoids together with the description of pyrethrinoid-resistant aphids has weakened insecticide-based control methods available to farmers. Since the deployment of insecticides in the 1980s, few research efforts were made to breed for rapeseed cultivars resistant to aphid-borne viral diseases. Thus, only few rapeseed cultivars released in Europe were reported to be TuYV-resistant, and the resistance phenotype of these cultivars was poorly characterized. In this study, several epidemiological parameters (infection rate, latency period, etc.) associated to the TuYV-resistance of the cv. Architect were estimated. Results showed a partial resistance phenotype for plants inoculated at the 2-/4-leaves stages and a resistance phenotype for plants inoculated at a more advanced growing stage. Moreover, analysis of infected plants highlighted (i) a poor quality of infected cv. Architect as a source of virus for transmission and (ii) an extended latency period for infected plants. Thus, dynamics of virus spread in the field should to be slower for Architect compared to susceptible rapeseed cultivars, which should lead to the maintenance of a higher proportion of healthy plants in the field.

## 1. Introduction

Most of the steps leading to virus infection of a susceptible host are possible thanks to the hijacking of the host’s cellular machinery by the virus. Due to the obligate parasite status of viruses, the main challenge for these pathogens is to optimize their access to a host in order to complete their biological cycle, to ensure the production of viral populations and to be able to transfer infectious particles from plant to plant. This parasitism induces disturbances in the biology of the host that results in mild to severe physiological alterations (e.g., yellowing, dwarfing, reddening, leaf deformation, stunting, and necrosis). For plants of agronomic interest, viral infection generally leads to yield reduction. Therefore, strategies have been developed to limit or to prevent the entry of viral entities into the compatible cellular environment of a susceptible host, and thus to block the infectious process at its early steps. These methods include the modification of growing practices (e.g., optimization of sowing dates [1,2] and management of volunteers [3]), the use of chemicals (e.g., insecticides for insect-borne viral diseases [4]), mineral oils, or bio-control products (e.g., plant extracts and micro-organisms [5]), and/or the use of genetic resources (i.e., resistant/tolerant cultivars [6]).

The identification of genotype(s) with a reduced level of susceptibility among the available genetic resources is the first step of breeding for resistance/tolerance. Phenotypic screening, supplemented by serological and molecular virus diagnostics, has made it possible to describe different levels of plant-virus incompatibility corresponding to extreme resistance (ER, non-host plant), hyper-sensitivity (HR, the virus is contained by the host in a necrotic zone consisting of a few cells at the inoculation site), partial resistance (the viral cycle is altered in its dynamics and/or efficiency), and tolerance (the virus multiplies in the host without disturbing plant physiology) (for a review, [7]). When a genetic resource presents such resistance/tolerance behavior, major efforts are made by breeders to introduce gene(s) involved in the phenotype into genetic background of cultivated susceptible varieties. However, this strategy could suffer from the scarcity of resistance/tolerance genes present in the available genetic resources.

Rapeseed (*Brassica napus*), one of the most important oil crops [8], can be infected by several viruses including turnip yellows virus (TuYV, Polerovirus genus, Luteoviridae family). TuYV is transmitted by aphids in a persistent, circulative, and non-propagative manner [9,10,11]. The peach-potato aphid *Myzus persicae* (Sulzer, 1776) is known to be the main vector of TuYV [10] while the cabbage aphid Brevicoryne brassicae (Linnaeus, 1758), commonly found in rapeseed fields, has been reported to be a poorly efficient TuYV vector [10]. The symptoms induced by TuYV include leaf reddening, interveinal chlorosis, and plant stunting. TuYV infection of susceptible rapeseed cultivars causes yield losses of up to 40% [12,13,14,15,16]. Thus, turnip yellows virus is one of the major threats for rapeseed production. The scarcity of TuYV-resistance sources in rapeseed germplasm and the low efficiency of control methods based on growing practices led farmers to use chemical strategies (i.e., insecticides) to limit the incidence of TuYV on their crops. Indeed, the development in the early 1980s of chemical solutions made it possible, through seed and/or foliar treatments, to protect treated areas against direct (feeding) and indirect (vectoring of pathogens from plant-to-plant within the field) impacts of insects. For more than twenty years, the use of neonicotinoid insecticides (NNI) was the main strategy to control TuYV, with nearly 25% of rapeseed cultivated areas in France grown with treated seeds [17]. However, insecticides could (i) induce side effects on the environment and on non-targeted organisms, and (ii) lead to the selection of insecticide-resistant individuals in aphid populations. In the context of the recent EU ban on the use of NNI in the field [18,19], which potentially increases crop exposure to insects and the viruses they transmit [20,21], the use of resistant/tolerant genotypes should be considered as the preferred solution to reduce the impacts of TuYV on rapeseed production.

The first genetic resource with the TuYV-resistance phenotype described in Brassica was the re-synthetized *B. napus* line ‘R54’ [22]. The ‘R54’ TuYV-resistance phenotype has been introgressed into several commercial varieties. However, it has been reported to be sensitive to elevated ambient temperature [23]. A recent work, carried out on accessions representing a subset of the *B. napus* genetic diversity, allowed the identification in the TuYV-resistant rapeseed cv. Yudal of a single dominant QTL on chromosome A04 that does not segregate with markers linked to the ‘R54’ resistance, suggesting independent origins of the TuYV resistances present in ‘R54’ and in cv. Yudal [24]. On the French market, the first rapeseed cultivar described for its TuYV-resistant phenotype was cv. Allison [Limagrain] in 2015. Since then, several cultivars (e.g., Architect [Limagrain], Angelico [Limagrain], Temptation [DSV], Coogan [RAGT], Smaragd [DSV], and Delice [DSV]) have been reported for their resistance to TuYV infection. However, no information is available on the impact of these resistant cultivars on the epidemiology of TuYV in fields. It is therefore necessary to study and describe steps of the infection process altered in a TuYV-resistant host in order to understand the impacts of resistant/tolerant cultivars on TuYV epidemics. Thus, different parameters that involved virus–host (infection rate, viral accumulation, and latency in infected plants) and vector–host (antibiosis and antixenosis) interactions have been identified as targets for this study. Using several experimental designs, these epidemiological parameters have been estimated for cv. Quizz ([RAGT], described to be tolerant to TuYV infection) and cv. Architect ([Limagrain], reported to be partially resistant to TuYV infection [25]) to determine whether and how these genotypes can participate in future strategies to control TuYV in a neonicotinoid-free agriculture.

## 2. Results

### 2.1. Infection Rate and Virus Accumulation in Plants

**Figure 1:**
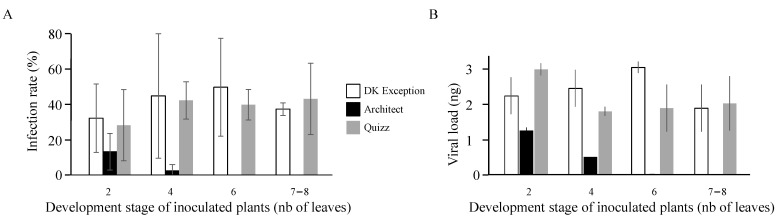
Infection rates of rapeseed genotypes (**A**) and virus load in infected plants (**B**) according to the development stage of plants at inoculation. Series of 20 plants at different development stages were inoculated with viruliferous (Turnip yellows virus, TuYV) aphids (2 aphids per plant) for 2 h inoculation access period. Three weeks after inoculation, the presence of TuYV in plants was tested by ELISA in the presence of standards corresponding to serially diluted fractions of purified TuYV-PS isolate. The positive threshold of the test was twice the OD_405_ value of healthy plant controls with a minimum value of OD_405_ = 0.1. The average infection rates (**A**) and the viral load in 100 µL of crude sap from infected plants (**B**) are presented. The experimental procedure was repeated at least twice for each genotype/developmental stage combination. The vertical bars represent the standard deviations associated with the data. At 4-leaves stage, only one out of the inoculated Architect plants was infected.

The susceptibility of rapeseed cultivars was assessed using a standardized inoculation protocol based on the use of viruliferous aphids (2 aphids/plant) maintained on test plants (at the 2-leaves stage) for 2 h inoculation access period (IAP). Analysis of sanitary status of plants 3 weeks after inoculation showed that this procedure leads to the infection of 32.4% (+/−19.4%) for the TuYV-susceptible referent cv. DK Exception (Figure 1A). This inoculation protocol was applied to cvs. DK Exception, Architect, and Quizz using plants at 2, 4, 6, and 7–8-leaves stages. Analysis of data showed that infection rates of cv. Quizz associated to the different growing stages were similar to data associated to cv. DK Exception (*p* > 0.272) while cv. Architect presented infection rates for 2-leaves (13.3% (+/−10.4%)) and 4-leaves (2.5% (+/−3.5%)) stages significantly lower than cvs. DK Exception and Quizz (*p* = 0.0323 and *p* = 1.55 × 10^−5^ for 2- and 4-leaves stages, respectively). Moreover, none of the cv. Architect plants inoculated at 6-leaves (*n* = 60) and 7–8 leaves (*n* = 60) stages were infected by TuYV, whereas the two other genotypes, for these two growing stages, were infected at a rate in the range 38–50% (Figure 1A). The use of serial diluted fractions of purified TuYV-PS isolate as standards in ELISA made it possible the quantification of viral load in infected plants (Figure 1B). Statistical analysis of these data showed significant differences for plants inoculated at 2-leaves stage (*p* = 0.037) with virus accumulation in cv. Architect (1.24 ng/100 µL plant sap) significantly lower (*p* = 0.022) than virus load of infected cv. DK Exception plants (2.22 ng/100 µL plant sap). Comparison of virus loads in infected cvs. Quizz and DK Exception for the four tested growing stages did not show significant differences (*p* > 0.19).

### 2.2. Fitness of Aphids

**Figure 2:**
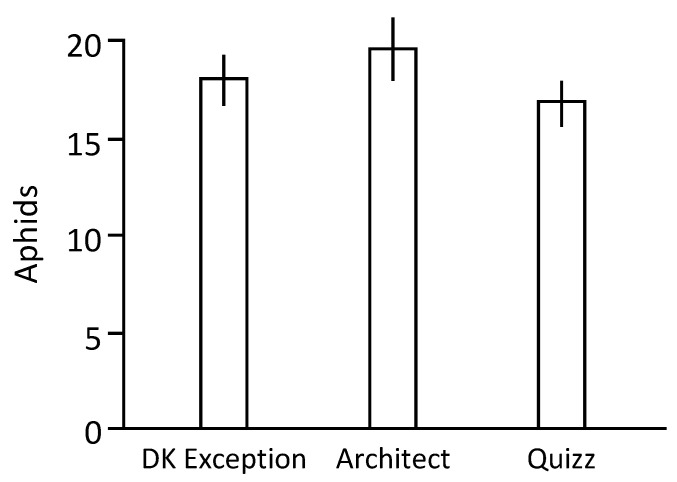
Number of aphids produced from a single L_1_ larvae of Mp34. Aphid larva L_1_ obtained from synchronized Mp34 population were placed on rapeseed plants in order to measure the size of the population produced in a 9 days period. The average population sizes obtained are presented. The experimental procedure, carried out on a series of 20 plants per genotype, was repeated 3 times. The vertical bars represent the standard deviations associated with the data.

The number of offspring produced by a single aphid maintained on a plant gives information on aphid–plant interactions. Under our experimental conditions, a single L_1_ larva of the Mp34 clone, sampled from a synchronized aphid population and maintained for 9 days on the reference cv. DK Exception, is able to produce 18.6 (+/−1.1) individuals (Figure 2). The number of aphids produced on cvs. Architect (19.7 (+/−1.3)) and Quizz (16.9 (+/−1.2)) did not allow to highlight significant differences between these rapeseed cultivars for this parameter (*p* = 0.2).

### 2.3. Aphid Dispersion and Virus Spread in Arena Tests

Dispersion of *M. persicae* and spread of TuYV were estimated using insect-proof cages containing 30 plants. Fourteen days after the release of a single viruliferous aphid (L_3_/L_4_ stage) in the middle of the cage, the aphid and its progeny were observed on about half of the plants (from 48% to 55%) with an average population density of adult morphs and nymphs of 7.6 to 12.7 individuals per plant (Table 1, columns a and b). The analysis of the ability of Mp34 to colonize plants in the arena shows a significant difference for the tested genotypes (*p* = 1.1 × 10^−2^). However, the number of individuals produced per colonized plant is similar for the three rapeseed cultivars (*p* = 0.501). The viruliferous status of the aphid released at the beginning of the experiment makes it possible to complete the observation of aphid dispersion by an analysis of the virus spread. Transmission rates calculated using healthy/infected status of either all plants of the arena (Table 1, column c; from 3% for cv. Architect to 42.4% for cv. Quizz) or only plants colonized by at least one aphid (Table 1, column d; from 4.8% for cv. Architect to 65.7% for Quizz) highlighted (i) the efficient spread of the virus in arena constituted by cv. DK Exception and cv. Quizz plants and (ii) the significant lower transmission efficiency obtained for cv. Architect (*p* = 2 × 10^−16^). The observation of aphids on plants at the end of the experiment does not allow to track all events that took place during the 14 days of the experiment. Indeed, during this period, plants can be visited by aphid(s) without the latter establishing a colony. Data showed that 10/67 (14.9%) cv. DK Exception and 19/112 (17%) cv. Quizz plants that were aphid-free at the end of the experiment were infected by TuYV (Table 1, column e). This indicates that at least one viruliferous aphid fed on these plants during the experiment. However, none of the 59 plants of cv. Architect described to be aphid-free at day 14 were infected.

### 2.4. Latency Period

**Figure 3:**
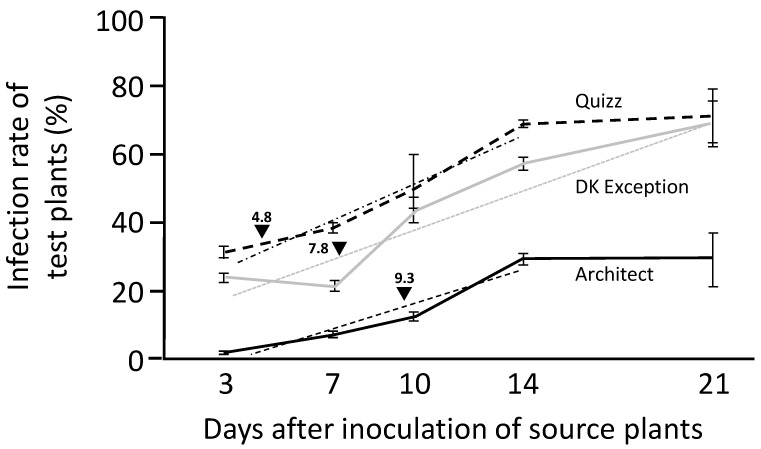
Transmission efficiency associated to source plants infected by TuYV for 3 to 21 days. Series of 8 plants (cvs. Architect, Quizz, and DK Exception) infected for 3, 7, 10, 14, and 21 days were used as sources for aphid-mediated transmission of TuYV-PS to susceptible cv. DK Exception test plants. Each source plant was used to inoculate ten test plants. Three weeks after inoculation, the presence of TuYV in test plants was tested by ELISA. Evolution of the quality of plants as a source for virus transmission are illustrated for each tested genotype. Linear regressions obtained from data from source plants infected for 3 to 14 days (cvs. Architect and Quizz) and for 3 to 21 days (cv. DK Exception) are drawn. The durations (expressed in days) of infection of source plants required to obtain 50% of the maximum infection rate are presented for each genotype by black triangles. The experimental procedure was repeated 3 times for each genotype. The vertical bars represent the standard deviations associated with the data.

Plant to plant transmissions carried out using source plants infected for a period ranging from a few days to several weeks provide access to latency period associated with virus/host interactions. However, such type of work requires to validate the infected status of all used source plants (especially those inoculated few days before being used as viral sources in the experiment). Thus, to study latency period of TuYV infection of rapeseed genotypes, the source plants used in the experiment were tested by RT-PCR at the end of the experiment (i.e., at least three weeks after inoculation (data not shown)) and all results associated with virus-free source plants were discarded from the data set. Under our experimental conditions, the use of cv. DK Exception source plants infected for 3 to 21 days led to the infection of cv. DK Exception test plants with rates from 24.8% (+/−1.1%) to 70.0% (+/−6.3%) (Figure 3). The transmission rates obtained for the cv. Quizz increase from 31.4% (+/−1.3%) for source plants infected for 3 days to 71.0% (+/−7.7%) for source plants infected for 21 days. Transmission rates obtained for this rapeseed cultivar are similar (Figure 3, D_3_, D_10_, and D_21_) or significantly higher (Figure 3, D_7_ (*p* = 3.2 × 10^−4^) and D_14_ (*p* = 1.4 × 10^−2^)) to cv. DK Exception plants, suggesting that infected cv. Quizz can be considered an equivalent or a better source of virus (i.e., at D_7_ and D_14_) than infected cv. DK Exception. The use of infected cv. Architect plants as viral sources in transmission experiments led to lower infection rates (*p* < 10^−7^) of cv. DK Exception test plants (from 1.9% (+/−0.2%) for source plants infected for 3 days to 29.3% (+/−7.6%) for source plants infected for 21 days). Finally, the transmission rates obtained for cvs. Quizz and Architect with source plants infected for 14 and for 21 days highlight maximum source values for virus spread which allow, under our experimental conditions, 71% (+/−6.3%) transmission efficiency for cv. Quizz and 29.3% (+/−7.6%) transmission efficiency for cv. Architect. For cv. DK Exception, the maximum source value for viral spread seemed not to be reached with source plants infected for 21 days. Linear increases of the source value of infected plants between the 3rd and 14th days after inoculation of cv. Quizz (r^2^ = 0.96) and cv. Architect (r^2^ = 0.92), and between the 3rd and 21st days after inoculation for cv. DK Exception (r^2^ = 0.90) were observed (Figure 3). Thus, transmission rates equivalent to half of those obtained with source plants at maximum source values, estimated using linear regressions, were obtained at 7.8, 4.8, and 9.3 days after inoculation for cvs. DK Exception, Quizz, and Architect, respectively (Figure 3).

## 3. Discussion

In Europe, insecticides of the neonicotinoid family are no longer available to farmers [18] and resistance to pyrethrinoids has been already described in numerous aphids species of economic importance including *M. persicae* [26]. Thus, the fine characterization of virus/aphid/rapeseed interactions involved in plant to plant transmission of TuYV appears to be essential for the implementation of future management strategies against this aphid-borne virus. The experiments carried out in this study allowed the analysis for different rapeseed cultivars of (i) viral infection key steps including infection rate, latency period, and quality of infected plants as sources for viral transmission, and (ii) aphid behavior including fecundity, antibiosis, and antixenosis phenomenon. The characterization of these important epidemiological parameters provides a better understanding of the processes that occur from the emergence of the first infected plant to the spread of TuYV in field, highlights the similarities between the cv. Quizz and the TuYV-susceptible cv. DK Exception (used as referent in this study) for their abilities to host aphids and virus, and accurately describes the partial TuYV-resistance phenotype carried by the rapeseed cv. Architect.

As turnip yellows virus is exclusively transmitted by aphids, the first step of the infection at the field scale corresponds to the introduction of the virus (primary infection) by viruliferous aphids flying from reservoirs. The low susceptibility of the cv. Architect compared to the TuYV-susceptible reference cv. DK Exception reduces the success of these primary infections by 2.4 and 18 times for inoculations occurring on plants at 2- and 4-leaves stages, respectively. Moreover, none of the cv. Architect plants inoculated at 6- and 7–8-leaves stages were infected under our experimental conditions, suggesting the expression of a resistance phenotype at these early growing stages. In France, the sowing period for rapeseed is from the end of August to the beginning of September. After sowing, four to fifteen days are needed for germinated seeds to produce cotyledons. Then, four to eight days later, the plantlets develop their first true leaf and quickly (at a rate of close to 2 new leaves per week) establish a rosette of about twenty-thirty leaves. Thus, under standard French growing conditions, the 6-leaves stage is reached by rapeseed plantlets at the 5th–6th weeks after sowing. Based on the resistance phenotype expressed from 6-leaves stage by cv. Architect, the latter is exposed to TuYV infections from the emergence of seedling to mid-October while the susceptible cv. DK Exception is exposed to virus infections for a longer period as illustrated by the infection rate (37.5–50%) obtained with plants inoculated at the 6- and 7–8-leaves stages. The intensity of aphid flights and the frequency of viruliferous aphids in autumn is known to vary in space and time [27]. Winged *Myzus persicae* migrate from the environment to rapeseed fields from few days after sowing (i.e., at the emergence of cotyledons) to the cold period of winter season. However, mild winter temperatures associated to climate change extend the period of aphid migration, increase the proportion of winged aphids in populations, stimulate aphid flight activity, and delay last winter flights [28,29]. Thus, growing the cv. Architect rapeseed with its reduced period of exposure to the risk of TuYV infection can be considered as a good strategy to limit viral incidence due to primary infections. However, primary infections usually represent only a small part of the overall epidemic process at the field scale. Indeed, the first infected plants (from primary infections) play important role in the spread (secondary infections) of the disease within the crop [30]. Thus, in addition to the introduction of viruliferous aphids in fields, the spread of the disease depends on both the population dynamics of aphids and the latency period of infected plants. The measures related to the monitoring of rapeseed/*M. persicae* interactions produced during this study did not reveal significant variation in the number of aphids produced after the maintenance of a single L_1_ larvae for 2 weeks on the tested rapeseed genotypes, suggesting that these genotypes are equivalent to host aphid populations. However, data indicate differences in the antixenosis phenomenon as denoted by the more efficient (1.14 times when compared to data associated to cv. DK Exception) colonization of cv. Architect plants by aphids in the arena-based experiments. This suggests that a field grown with cv. Architect can be associated to a higher proportion of plants infested by aphids, and consequently could be more exposed to plant-to-plant transmissions of aphid-borne virus.

Viruliferous winged aphids are able to introduce TuYV into the field by feeding on visited plants. TuYV is transmitted in a persistent non-propagative manner [11]. To initiate the infection, TuYV particle(s) must be introduced in the phloem of a susceptible host. Thus, success of TuYV transmission depends on feeding behavior of aphids (i.e., characteristics of phloem punctures) which could be different between host genotypes. Each individual of the progeny produced by an adult aphid (viruliferous or not) is virus-free until it has the opportunity to feed on an infectious plant. Thus, the quality of an infected plant as a source of virus for aphid-mediated transmission and the duration of the latency period (delay for an infected plant to acquire the infectious status) directly influence the ability of virus-free aphids (born in the field or flying from outside) to acquire and spread the disease from primary infected plants. Results showed that cv. Architect is a poor viral source for transmission (maximum source value of infected rapeseed plants is 2.3 times lower for cv. Architect than for cv. DK Exception) and infected cv. Architect plants require a longer period to acquire the infectious status (mid-duration of the latency period is 1.5 days longer when compared to cv. DK Exception). Taken together with the lower TuYV-susceptibility of cv. Architect described in this study, these epidemiological parameters can partly explain the low prevalence of infected cv. Architect plants reported in the arena test carried out in this work (3% (+/− 0.5%) infected cv. Architect plants vs. 38.5% (+/−3.2%) infected cv. DK Exception plants) and in data from field trials performed under low, standard and high virus pressures [25].

This fine characterization of interactions between members of the turnip yellows pathosystem allows to conclude that the partial TuYV-resistance of cv. Architect plants reduces the efficiency of both primary infections due to the migration of viruliferous aphids that occurs in autumn (lower infection rate and resistance phenotype expressed from the 6-leaves stage) and secondary spread of the disease in fields (longer latent period and lower source value for infected plants). These characteristics, which allow the maintenance of a greater proportion of healthy plants in the plots by targeting several steps of the epidemiological process at the field scale, make cv. Architect an interesting genetic support for the development and/or optimization of future strategies to control TuYV in rapeseed production. However, to complete the characterization of the partial resistance phenotype of cv. Architect, it remains important (i) to evaluate the behavior of this genotype against viral pressures differing in intensity, frequency, and duration (reflecting the multiple flights of winged aphids in autumn), and (ii) to test its durability against the genetic diversity and the evolution of TuYV. This obviously will be the next steps of our work on TuYV/*M. persicae*/rapeseed cv. Architect interactions.

## 4. Materials and Methods

### 4.1. Plants, Insects, and Virus

Rapeseed cvs. DK Exception (susceptible to TuYV), Architect (described for its TuYV-resistant phenotype), and Quizz (described for its TuYV-tolerant phenotype) were used in the experiments. Seeds were sown in N2 soil (Neuhaus^®^ Huminsubstrat N2, Klasmann-Deilmann, Geeste, Germany) and maintained in a sowing chamber (day/night: 12 h/12 h, 23 °C/20 °C) for five days. Then, seedlings were transferred either individually in pots (TEKU^®^ Pöppelmann France S.A.S., Rixheim, France) 7 × 7 cm and 9 × 9 cm or in pools of 30 plants in trays (L × W × H: 30 × 25 × 7 cm) depending on the experiments. Plantlets were grown in a growth chamber (day/night: 16 h/8 h, 25 °C/20 °C) for 7 days before being used in the experiments.

The *Myzus persicae* clone Mp34 [31] was used for virus transmissions. Virus-free Mp34 was maintained in a growth chamber in small plexiglass cages in the presence of healthy rapeseed plants cv. DK Exception.

Isolate PS of turnip yellows virus (TuYV-PS, collected in department of Ain, France, 2018) was maintained on rapeseed cv. DK Exception plants in small plexiglass cages in the presence of Mp34.

### 4.2. Infection Rates

Plantlets at different growing stages (2, 4, 6, and 7–8 leaves) were used to assess the susceptibility of rapeseed genotypes to TuYV-PS. Using a brush, viruliferous aphids (L_2_/L_4_ stages; 2 aphids/plant) were deposited on test plants. Then, plants were covered with micro-perforated plastic bags. At the end of the inoculation access period (i.e., 2 h), the viruliferous aphids were manually removed from plants and the latter were treated with an insecticide (Pirimor^®^ 0.1% *v*/*v*, Syngenta^®^, Basel, Switzerland) before being transferred in a growth chamber. Three weeks later, plants were sampled and the presence of TuYV in each plant was assessed by enzyme-linked immuno-sorbent assay (ELISA). This complete procedure was replicated at least twice using sets of 20 plants/genotype/replication.

### 4.3. Production of Aphid Colonies

Larvae of *M. persicae* Mp34 were obtained from synchronized progenies produced on rapeseed cv. DK Exception. L_1_ larvae were deposited on test plants at 2-leaves stage (one larva/plant). Then, plants were individually covered by micro-perforated plastic bags and kept in a growth chamber for 9 days. At the end of this period, the number of aphids present on each plant was counted. This procedure was replicated 3 times using sets of 20 plants/genotype/replication.

### 4.4. Arena Tests

Thirty rapeseed plantlets (at 2-leaves stage), planted in 6 rows of 5 plants each (in trays (L × W × H: 30 × 25 × 7 cm)), constitute the arena design used in the experiment. L_3_/L_4_ larvae of viruliferous Mp34 were deposited (1 larva/arena) at the center of the arena. The whole arena (i.e., the 30 plants and the single aphid) was covered by insect proof net and maintained in a growth chamber for 14 days. At the end of this period, the number of aphids present on each plant was counted. Then, plants were sprayed with insecticide (Pirimor^®^ 0.1% *v*/*v*), maintained in the growth chamber for 3 weeks, and individually tested for the presence of TuYV by ELISA. This experimental procedure was carried out with 2 arenas per genotype and replicated 4 times.

### 4.5. Latency Period of Infected Plants

Viruliferous Mp34 aphids (L_2_/L_4_ stages) were deposited (5 aphids/plant) for 24 h on healthy rapeseed plantlets (cv. DK Exception, Architect and Quizz) at two-leaves stage. At the end of this inoculation access period (IAP), the aphids were manually removed from plants. These aphid-free TuYV-inoculated plants were considered in the experiment as ‘source’ plants. At 5 dates after inoculation of source plants (i.e., at 3, 7, 10, 14, and 21 days), non viruliferous Mp34 aphids (L_1_/L_4_ stage, at least 30 aphids/source plant) were contained for 24 h acquisition access period (AAP) on source plants. At the end of AAP, aphids from each source plant were transferred to healthy cv. DK Exception plants (2 aphids/plant) for 24 h IAP. These plants were considered as ‘test’ plants. At the end of IAP, test plants were treated with Pirimor^®^ (0.1% *v*/*v*). All source and test plants were maintained in a growth chamber for 3 weeks after the end of their respective IAP. Then, plants were tested by ELISA (test plants) or RT-PCR (source plants) for the presence of TuYV (see below for diagnostic procedure). This procedure was carried out with 8 TuYV-infected source plants for each genotype/date combination and 10 test plants/source plant. The whole experiment was replicated 3 times.

### 4.6. Serological and Molecular Detection of TuYV in Plants

Each plants (i.e., all leaves) sampled for ELISA [32] was ground using a Pollähne press (MEKU^®^, Wennigsen, Germany). Plant material sampled for RT-PCR (i.e., pools of leaf discs from each leaf) was placed in sterile microtubes containing 1 metal ball (4 mm in diameter) and then ground in the presence of liquid nitrogen using the MM301 mill (Retsch, Hann. Münden, Germany).

For ELISA, wells of a microtiter plate (NUNC, Maxisorp) were incubated at 37 °C for 4 h with polyclonal anti-TuYV antibodies (LOEWE^®^, Sauerlach, Germany) previously diluted (1/400 (*v*/*v*)) in carbonate buffer (15 mM Na_2_CO_3_, 35 mM NaHCO_3_, pH = 9.6). Between each step of the ELISA protocol, the plates were washed 3 times with PBST buffer (137 mM NaCl, 8 mM Na_2_HPO_4_, 12H_2_O, 2,7 mM KCl, 1.5 mM KH_2_PO_4_, 0.05% (*v*/*v*) Tween 20), 2% (*w*/*v*) polyvinylpyrrolidone 40T). One hundred µl of plant sap was deposited in the wells and incubated overnight at 4 °C. Alkaline phosphatase coupled antibody (100 µL) diluted (1/400 (*v*/*v*)) in conjugated buffer (PBST buffer, 2% (*w*/*v*) ovalbumin) was deposited in wells and incubated for 4 h at 37 °C. After a final wash, 100 µL of diethanolamine (1N, pH = 9.8) containing p-nitrophenylphosphate (1 mg/mL) was deposited in wells and plate was incubated at room temperature and in the dark for 2 h. Then, optical density of each well was measured at 405 nm (OD_405_) using a spectrophotometer (Multiskan™ FC; Thermo Scientific™, Waltham, Massachusetts, USA). The positive threshold of the test was twice the OD_405_ value of healthy plant controls with a minimum value of OD_405_ = 0.1. A serial dilution of a semi-purified TuYV-PS fraction (749 ng/µL) was prepared and used in each plate to make it possible the transformation of raw OD_405_ values in viral load in tested samples.

Total RNA from rapeseed leaves was extracted using the Monarch^®^ Total RNA Miniprep Kit (New England Biolabs^®^, Ipswich, MA, USA). The presence of TuYV in 1 µL RNA fraction was tested using the OneTaq^®^ One-step RT-PCR Kit (New England Biolabs^®^) according to the manufacturer’s recommendations, a melting temperature of 55 °C, and the primers CPBM+ (5’-atgaatacggtcgtgggtaggag-3’) and CPBM- (5’-ccagctatcgatgaagaaccattg-3’) [33]. After amplification, 5 µL of the reaction mixture was analyzed by agarose gel electrophoresis (1.5% *w*/*v*). Amplified products were revealed in the presence of ethidium bromide using a transilluminator.

### 4.7. Statistical Analyses

Statistical analyses were performed using R version 3.6.0 [34]. The numbers of infected and healthy plants were analyzed using a quasibinomial generalized linear model (GLM). The effects were tested by comparing nested models using a Student test (drop1 function). The comparison of the viral loads was performed by using the Kruskal–Wallis test.

## Figures and Tables

**Table 1:** Monitoring of aphids on plants in arena tests.

Genotype	Colonisation of Plants (%) ^a^	Aphids/Plant ^b^	Transmission Rate (%)		
			In the Arena ^c^	For Plants Colonized by Aphids ^d^	For Aphid-Free Plants ^e^
DK Exception	48.0 (+/−6.3)	12.7 (+/−2.0)	35.8 (+/−3.2)	62.9 (+/−4.2)	14.9 (10/67)
Quizz	49.1 (+/−3.9)	9.1 (+/−0.5)	42.4 (+/−2.6)	65.7 (+/−1.7)	17.0 (19/112)
Architect	54.7 (+/−6.1)	7.6 (+/−0.7)	3.0 (+/−0.5)	4.8 (+/−0.9)	0.0 (0/59)

^a^: Percentage of plants colonized by at least one aphid at the end of the experiment (i.e., 14 days after the introduction of the viruliferous aphid in the arena). ^b^: Plants colonized by at least one aphid at the end of the experiment were considered to calculate this value. ^c^: Transmission rate based on the total number of plants present in the arena (i.e., 30 plants). ^d^: Transmission rate based on the number of plants colonized by at least one aphid at the end of the experiment. ^e^: Aphid-free plants from all replicates were used to calculate this percentage. Date presented in columns ^a,b,c^ and ^d^ are in percentage (+/− standard error). Data presented in column ^e^ are in percentage (number of plants with aphid(s)/number of plants not colonized by aphids).
